# Congenital lung abnormalities on magnetic resonance imaging: the CLAM study

**DOI:** 10.1007/s00330-023-09458-7

**Published:** 2023-02-24

**Authors:** Bernadette B. L. J. Elders, Casper M. Kersten, Sergei M. Hermelijn, Piotr A. Wielopolski, Harm A. W. M. Tiddens, J. Marco Schnater, Pierluigi Ciet

**Affiliations:** 1 Department of Paediatric Pulmonology and Allergology, Erasmus MC - Sophia Children’s Hospital, University Medical Centre Rotterdam, Rotterdam, The Netherlands; 2 Department of Radiology and Nuclear Medicine, Erasmus MC, University Medical Centre Rotterdam, Rotterdam, The Netherlands; 3 Department of Paediatric Surgery, Erasmus MC - Sophia Children’s Hospital, University Medical Centre Rotterdam, Rotterdam, The Netherlands; 4 Radiology Department, University of Cagliari, Cagliari, Italy

**Keywords:** Congenital lung abnormalities, Imaging, MRI, CT, Paediatric

## Abstract

**Objectives:**

Follow-up of congenital lung abnormalities (CLA) is currently done with chest computer tomography (CT). Major disadvantages of CT are exposure to ionizing radiation and need for contrast enhancement to visualise vascularisation. Chest magnetic resonance imaging (MRI) could be a safe alternative to image CLA without using contrast agents. The objective of this cohort study was to develop a non-contrast MRI protocol for the follow-up of paediatric CLA patients, and to compare findings on MRI to postnatal CT in school age CLA patients.

**Methods:**

Twenty-one CLA patients, 4 after surgical resection and 17 unoperated (mean age 12.8 (range 9.4–15.9) years), underwent spirometry and chest MRI. MRI was compared to postnatal CT on appearance and size of the lesion, and lesion associated abnormalities, such as hyperinflation and atelectasis.

**Results:**

By comparing school-age chest MRI to postnatal CT, radiological appearance and diagnostic interpretation of the type of lesion changed in 7 (41%) of the 17 unoperated patients. In unoperated patients, the relative size of the lesion in relation to the total lung volume remained stable (0.9% (range − 6.2 to + 6.7%), *p* = 0.3) and the relative size of lesion-associated parenchymal abnormalities decreased (− 2.2% (range − 0.8 to + 2.8%), *p* = 0.005).

**Conclusion:**

Non-contrast-enhanced chest MRI was able to identify all CLA-related lung abnormalities. Changes in radiological appearance between MRI and CT were related to CLA changes, patients’ growth, and differences between imaging modalities. Further validation is needed for MRI to be introduced as a safe imaging method for the follow-up of paediatric CLA patients.

**Key Points:**

*• Non-contrast-enhanced chest MRI is able to identify anatomical lung changes related to congenital lung abnormalities, including vascularisation.*

*• At long-term follow-up, the average size of congenital lung abnormalities in relation to normal lung volume remains stable.*

*• At long-term follow-up, the average size of congenital lung abnormalities associated parenchymal abnormalities such as atelectasis in relation to normal lung volume decreases.*

**Supplementary information:**

The online version contains supplementary material available at 10.1007/s00330-023-09458-7.

## Introduction

In recent years, the incidence of prenatally diagnosed congenital lung abnormalities (CLA) has increased fourfold up to 4 cases per 10,000 life-births, mostly due to improved prenatal screening [1]. Different types of CLA can be distinguished: congenital pulmonary airway malformation (CPAM), bronchopulmonary sequestrations (BPS), hybrid lesions (i.e. features of both CPAM and BPS), congenital lobar overinflation (CLO), bronchogenic cyst (BC) and bronchial atresia (BA) [2–4]. CLA can cause symptoms, such as respiratory distress, recurrent infections, and failure to thrive, but are asymptomatic in around 70–80% of the cases [2, 5]. While symptomatic CLA are treated with surgical resection, controversy exists on the correct management of asymptomatic CLA, as highlighted by several surveys carried out in Europe, the UK, and Canada [5–9]. These different approaches create management dilemmas. In case a wait-and-see management is preferred, it remains unclear how and when these patients should undergo imaging to monitor changes in the CLA configuration or to detect possible malignant degeneration [3, 10]. The current gold standard for postnatal CLA imaging is chest computed tomography (CT), but this has disadvantages, such as exposure to ionizing radiation and need for a contrast agent to visualise vascularisation of the lesion [11–13]. Magnetic resonance imaging (MRI) could be a safe alternative, being free of ionizing radiation and comparable at showing lung structure, as recently seen in a postnatal cohort of CLA patients [14, 15]. To date, no studies on the long-term follow-up of CLA using MRI have been reported.

Our hypothesis was that chest MRI can be used for the long-term follow-up of CLA patients; and therefore, the objective of this study was to develop and test a non-contrast chest MRI protocol for the follow-up of paediatric CLA patients, and to compare MRI to postnatal CT findings in a cohort of school-age CLA patients.

## Methods

**Fig. 1:**
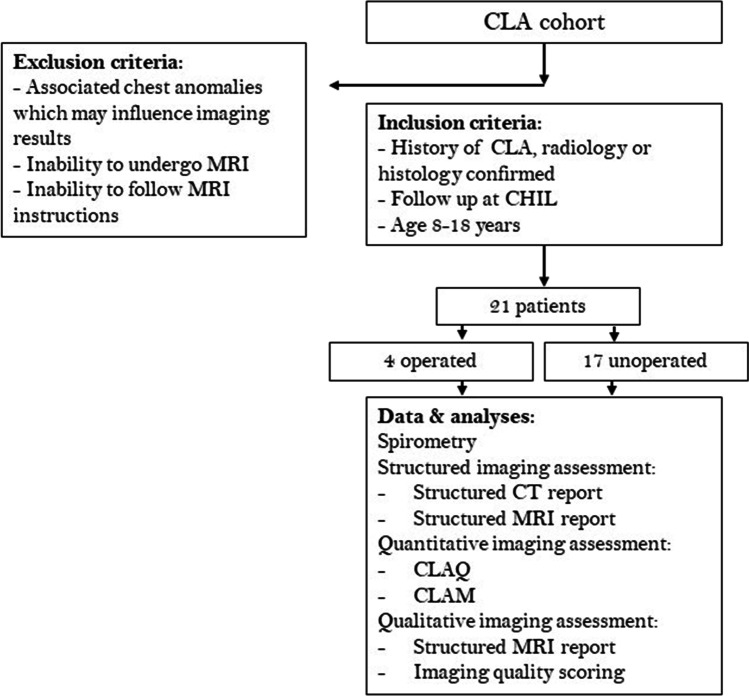
Flowchart of the study design and included patients. CHIL, surgical long-term follow-up outpatient clinic; CLA, congenital lung abnormality; CT, computed tomography; MRI, magnetic resonance imaging

A flowchart of the study design and patient inclusions is shown in Fig. 1. Patients from the surgical long-term follow-up program with a radiologically or histologically (in case of surgical resection) confirmed CLA, aged between 8 and 18 years, were approached to participate in this study [16]. Patients with associated thoracic anomalies which could alter imaging results (e.g. interstitial lung diseases, cystic fibrosis, bronchopulmonary dysplasia or pulmonary hypertension), and patients with contraindications to undergo MRI or unable to follow instruction during the MRI were excluded from participation. The study was approved by the local medical ethics committee (MEC2018-107); written informed consent was obtained from all patients and/or legal representatives.

Data on primary diagnosis, symptoms, and surgical resection were retrieved from the electronic patient dossier. All patients underwent spirometry and chest MRI. Postnatal CTs were collected to compare with the MRIs.

### Spirometry

Spirometry was completed in all patients according to European Respiratory Society (ERS)/American Thoracic Society (ATS) guidelines and data are presented as percentage predicted and *z*-scores as reported by the General Lung function Institute (GLI) [17–19].

#### CT

A free breathing contrast-enhanced chest CT scan was acquired within the first year of life using a standardised protocol. The majority of scans (18/21) were acquired on an Emotion 6 CT scanner (Siemens Healthcare), 2/21 were acquired on a SOMATOM Definition CT scanner (Siemens Healthcare), and 1/21 was acquired on a Lightspeed QX/i CT scanner (GE Healthcare). The following range of parameters applies, with differences caused by changes in CT protocols over the years: tube load 20–101 mAs, kilovoltage peak 80–120 kV, field-of-view 103–214 mm, slice thickness 0.75–2.5 mm, kernel B30s–B75f. In 90% of patients, a split bolus contrast enhancement protocol was used with both arterial and venous contrast enhancement.

#### MRI

All patients underwent a spirometry-guided chest MRI on a 1.5-Tesla scanner (Artist, GE Healthcare). The MRI protocol consisted of sagittal 3D SPGR proton density weighted (PD-w) sequences during end-inspiratory and expiratory breath-hold, an axial 2D PROPELLER fat-suppressed (FS) T2-weighted (T2-w) sequence during free-breathing, and a 3D axial ZTE PD-w sequence during free breathing. To minimalise scan time, only in those patients with known vascular abnormalities on postnatal CT, a coronal non-contrast-enhanced MR angiography (MRA) 3D FIESTA T2/T1-w sequence was added (online supplement 1). Total scan time was 30 min.

### Structured imaging assessment

Structured radiological reports describing findings on postnatal CTs and school-age MRIs (online supplement 2) were completed [3]. CLA types were defined as follows: CPAM, cystic abnormality without systemic arterial blood supply; BPS, solid lesion with systemic arterial blood supply; hybrid lesion, combination of CPAM and BPS; BC, (partial) fluid-filled cyst near the mediastinum; CLO, overinflated hypodense lung lobe; BA, a focal interruption of a lobar, segmental, or subsegmental bronchus with associated peripheral mucus impaction (bronchocèle, mucocèle) and associated hyperinflation of the obstructed lung segment [3]. All scans were anonymised and randomly ordered, after which they were assessed by an observer with 10 years’ experience in thoracic radiology (P.C.). There was a 4-week interval between the CT and MRI scoring to prevent recall bias. A comparison was made between the abnormalities seen on postnatal CT and school-age MRI, and their diagnostic interpretation.

### Quantitative imaging assessment

**Fig. 2:**
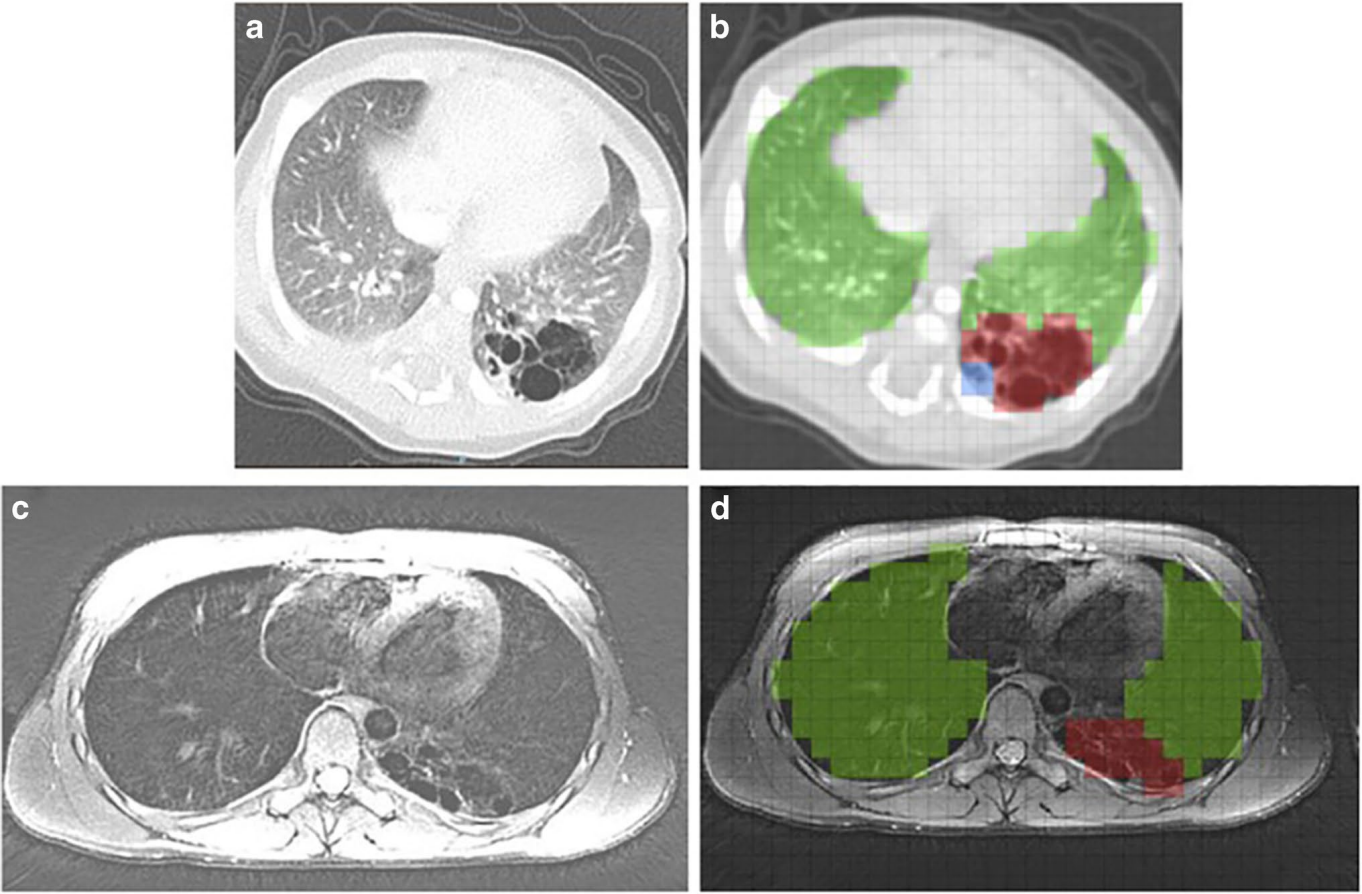
Example of an axial postnatal CT (**a**) and school-age MRI (**c**) in a patient with a CPAM of the left lower lobe. Both images show a multicystic, air-filled lesion. CLAQ scoring (**b**) shows normal lung tissue (green), lesion-associated parenchymal abnormalities (atelectasis, blue) and the lesion (red); the CLAM scoring (**d**) shows normal lung tissue (green) and the lesion (red)

To compare the relative volume of the lesion between postnatal CT and school-age MRI, we computed the volume of CLA lesion and associated parenchymal abnormalities, and the volume of normal lung tissue in relation to total lung volume. Volume quantification was done with a morphometry-based quantitative grid system using an in-house developed software [20–22]. A simplified version of the previously published Congenital Lung Abnormalities Quantification on CT (CLAQ) scoring method was used to score postnatal CTs [20]. For the school-age MRIs, a similar scoring method was used: the Congenital Lung Abnormalities quantification on MRI (CLAM) scoring method. For both the CLAQ and the CLAM scoring methods, equidistant axial images with a maximum distance of 3 mm between the slices were overlaid with a grid and each grid was scored hierarchically according to the abnormality within. Three categories were scored in which the highest hierarchy was assigned to lesional abnormalities followed by lesion-associated parenchymal abnormalities defined as parenchymal hypo- or hyperdensity and normal lung tissue. In addition, on the axial-reconstructed MR images in end- expiration, grids were hierarchically scored for hypo-intense regions and normal lung tissue. From the grid scoring, the software calculates a volume and relative percentage of the lesion, lesion-associated parenchymal abnormalities, and normal lung tissue. Figure 2 shows an example of both the CLAQ and CLAM scoring methods. The CLAQ and CLAM were scored on anonymised and randomly ordered scans by two certified observers (S.H. and B.E.) with 2 and 4 years’ experience in thoracic imaging, respectively. Both observers completed a standardised training module that included scoring four practice batches of five CT scans each, including all types of CLA to assess performance. For the CLAM scoring, the highest percentage of either the lesion or lesion-associated parenchymal abnormalities as seen on any of the sequences is presented, since not all abnormalities are equally visualised on each sequence due to different MR weighting [23].

The CLAQ and CLAM outcomes were compared to evaluate change in the relative size of the CLA lesion, lesion-associated parenchymal abnormalities, and normal lung tissue between postnatal CT and school-age MRI.

### Qualitative imaging assessment

To assess whether MRI is able to visualise all CLA-related abnormalities and to determine which sequences are best for this aim, a qualitative imaging assessment was performed. Firstly, the structured MRI report contained questions on whether certain CLA-related abnormalities were visible on MRI. Secondly, the structured MRI report contained questions on which sequences were best to visualise certain abnormalities (online supplement 2B). Thirdly, a qualitative scoring was applied on all MR images according to an adjusted version of the scoring method proposed by Bae et al (online supplement 3) [24]. In short, all MRI sequences were scored on the depiction of fissures, intrapulmonary vessels and bronchi, the presence of noise/artefacts, and overall acceptability, grading from unacceptable to superior on a 5-point scale.

### Statistics

Data are presented as mean ± standard deviation for parametric data and median (range or interquartile range) for non-parametric data. SPSS Statistics (version 25, IBM SPSS) was used for the data analysis. Data comparison was done using the parametric *t*-test for normally distributed data and the Mann–Whitney *U* test for non-normally distributed data. We assumed a 5% significance level.

## Results

Patient inclusions are shown in Fig. 1; characteristics and spirometry outcomes of all study participants are shown in Table 1. In total, 21 CLA patients were included with a mean age of 12.8 (range 9.4–15.9) years. The most common CLA was CPAM (*n* = 10, 48%). Four patients underwent surgical resection of whom two patients with a CPAM were operated on because of development of symptoms (respiratory distress and/or cardiac volume overload). Furthermore, two patients with a BPS were operated on because of respiratory distress and recurrent infection, respectively.

**Table 1:** Characteristics and spirometry results of all study participants

	All patients (*n* = 21)	Unoperated (*n* = 17)	Operated (*n* = 4)
Age at MRI (years)	12.8 (9.4–15.9)	12.6 (9.4–15.9)	13.8 (11.3–15.9)
Gender (% female)	52	47	75
Type of CLA (%)			
CPAM	48	47	50
BPS	33	29	50
Hybrid lesion	10	12	0
Bronchial atresia	19	24	0
Age at surgery (months)	-	-	2 (1–30)
Reason for surgery (*n*)			
Respiratory distress	-	-	3
Infection	-	-	1
Volume overload	-	-	1
Type of surgery (*n*)			
Pneumonectomy	-	-	1
Lobectomy	-	-	1
Lesion resection	-	-	2
FVC			
% predicted	95.6 ± 12.9	98.8 ± 10.7	82.0 ± 13.8
z-score	− 0.38 ± 1.1	− 0.10 ± 0.9	− 1.58 ± 1.2
FEV_1_			
% predicted	91.2 ± 15.6	94.7 ± 12.5	76.8 ± 21.3
*z*-score	− 0.74 ± 1.3	− 0.45 ± 1.0	− 1.94 ± 1.7
FEV_1_**/**FVC			
% predicted	95.0 ± 8.9	95.7 ± 7.9	92.0 ± 13.6
*z*-score	− 0.69 ± 1.0	− 0.65 ± 0.9	− 0.87 ± 1.5
PEF			
% predicted	94.9 ± 17.9	98.0 ± 15.5	81.8 ± 23.9
*z*-score	− 0.44 ± 1.1	− 0.24 ± 0.9	− 1.30 ± 1.7
FEF_25_			
% predicted	94.5 ± 20.7	98.9 ± 16.7	76.0 ± 28.6
*z*-score	− 0.50 ± 1.4	− 0.24 ± 0.9	− 1.73 ± 2.1
FEF_50_			
% predicted	81.5 ± 23.7	83.4 ± 21.8	73.8 ± 33.6
*z*-score	− 1.41 ± 1.6	− 1.29 ± 1.5	− 1.95 ± 2.5
FEF_75_			
% predicted	81.7 ± 29.1	84.5 ± 28.4	69.8 ± 33.6
*z*-score	− 0.73 ± 1.1	− 0.61 ± 1.0	− 1.23 ± 1.4
MEF_75/25_			
% predicted	81.2 ± 23.8	84.2 ± 22.2	68.5 ± 30.0
*z*-score	− 0.95 ± 1.2	− 0.79 ± 1.1	− 1.61 ± 1.6
VCmax			
% predicted	96.7 ± 12.6	99.2 ± 10.5	82.7 ± 16.9
*z*-score	− 0.30 ± 1.1	− 0.08 ± 0.9	− 1.55 ± 1.5

*BPS*, bronchopulmonary sequestration; *CPAM*, congenital pulmonary airway malformation; *CLA*, congenital lung abnormality; *FEF*_*25,50,75*_, forced expiratory flow at 25, 50, and 75% of expiration; *FEV*_*1*_, forced expiratory volume in 1 s; *FVC*, forced vital capacity; *MEF*, mean expiratory flow; *MRI*, magnetic resonance imaging; *PEF*, peak expiratory flow; *VCmax*, maximum vital capacity

Data are presented as mean ± standard deviation or median (range)

All patients successfully completed the MR examination. Median interval between postnatal CT and school-age MRI was 12.1 (range 10.0–14.7) years.

### Spirometry

Spirometry was within the normal range for all patients (*z*-score for all measurements between − 1.41 and − 0.30). Patients with a history of surgical resection had lower *z*-scores on spirometry (*z*-scores for all measurements between − 1.95 and − 0.87). However, this was mostly caused by one patient with a history of a left pneumonectomy with spirometry *z*-scores of − 2.15 (FVC), − 3.77 (FEV_1_), and − 2.98 (FEV_1_/FVC).

### Structured qualitative assessment

**Fig. 3:**
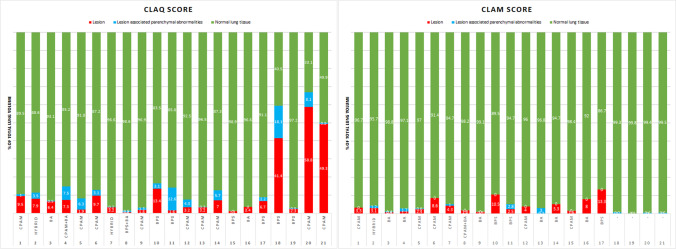
Overview of the change in percentage of lung affected by the CLA on postnatal CT compared to school-age MRI

**Fig. 4:**
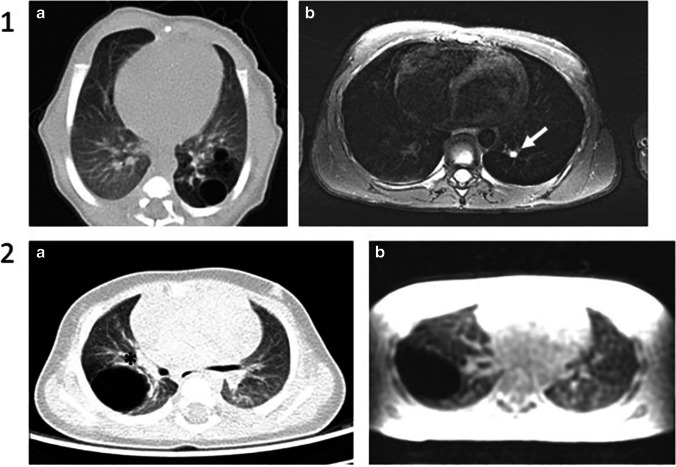
Example images of changed appearance of CLA between postnatal CT and school-age MRI. Patient 1: axial postnatal CT showing an air-filled multicystic CPAM and BA in the left lower lobe (**a**) and an axial T2-weighted PROPELLER image at school-age showing only BA in the left lower lobe (arrow) (**b**). Patient 2: axial postnatal CT showing an air-filled CPAM surrounded by lesion-associated parenchymal atelectasis (asterisk) (**a**) and an axial SPGR expiratory image at school-age showing an air-filled CPAM without lesion-associated parenchymal abnormalities (**b**)

Results from the structured CT and MRI reports are shown in Tables 2 and 3, and Fig. 3. Between postnatal CT and school-age MRI, the appearance and diagnostic interpretation of the type of CLA changed in 7 patients of the unoperated group (41%). In three patients, the lesion was classified as a CPAM on postnatal CT and as a BA on school-age MRI, and in one patient a mixed lesion (CPAM and BA) was seen on postnatal CT, and only a BA was found on school-age MRI. In addition, in three patients, the solid component of the lesion classified as a hybrid lesion (*n* = 1) or BPS (*n* = 2) appeared as cystic tissue on school-age MRI. Example images of changing appearances of CLA between postnatal CT and school-age MRI are shown in Fig. 4.

**Table 2:** Overview of the quantitative scoring of the postnatal CT and school-age MRI per patient

Patient	CLAQ				CLAM				
						Free**-**breathing MRI			Expiratory MRI
	Diagnostic interpretation	Lesion	Lesion**-**associated parenchymal abnormalities	Normal lung tissue	Diagnostic interpretation	Lesion	Lesion**-**associated parenchymal abnormalities	Normal lung tissue	Hypointense regions
	Unoperated								
1	CPAM	9.5	1.0	89.5	CPAM	3.3	0	96.7	7.2
2	Hybrid lesion	7.9	3.5	88.6	Hybrid lesion	3.1	1.2	95.7	5.7
3	BA	6.4	0.5	93.1	BA	0.8	0.4	98.8	0
4	CPAM + BA	7.3	7.5	85.2	BA	1.2	1.7	97.1	24.3
5	CPAM	1.9	6.3	91.8	CPAM	2.4	0.6	97.0	0.2
6	CPAM	9.7	3.1	87.2	CPAM	8.6	0	91.4	11.8
7	Hybrid lesion	3.2	0.2	96.6	CPAM	4.3	1.0	94.7	7.3
8	BPS + BA	0.5	0.9	98.6	CPAM + BA	1.8	0	98.2	0
9	CPAM	1.8	1.3	96.9	BA	0.9	0	99.1	3.3
10	BPS	13.4	3.1	83.5	BPS	10.5	0	89.5	17.9
11	BPS	1.6	12.6	85.8	BPS	2.5	2.8	94.7	10.7
12	CPAM	3.2	4.3	92.5	CPAM	4.0	0	96.0	5.3
13	CPAM	3.2	0.3	96.5	BA	0.2	3.0	96.8	5.0
14	CPAM	7.0	5.7	87.3	BA	5.3	0	94.7	7.7
15	BPS	1.1	0	98.9	CPAM	1.6	0	98.4	0
16	BA	3.4	0	96.6	BA	8.0	0	92.0	17.2
17	BPS	6.7	2.2	91.1	BPS	13.3	0	86.7	12.5
	Operated								
18	BPS	41.4	18.1	40.5	-	0	0.7	99.3	4.5
19	BPS	2.3	0.5	97.2	-	0	0.2	99.8	0
20	CPAM	58.8	8.1	33.1	-	0	0.6	99.4	0
21	CPAM	49.3	0.8	49.9	-	0	0.5	99.5	23.8

*BA*, bronchial atresia; *BPS*, bronchopulmonary sequestration; *CLAQ*, congenital lung abnormalities quantification on computed tomography; *CLAM*, congenital lung abnormalities quantification on magnetic resonance imaging; *CPAM*, congenital pulmonary airway malformation

Data are presented as % of total lung volume

**Table 3:** CLAQ and CLAM scoring results from all unoperated patients

	CLAQ (*n* = 17)	CLAM (*n* = 17)	Change in abnormalities	*p *value
Age (years)	0.3 (0.0–0.8)	12.3 (9.4–15.9)	11.9 (9.1–15.9)	-
Free breathing				
Lesion	3.4 (0.5–13.4)	3.1 (0.2–13.3)	− 0.9 (− 6.2 to + 6.7)	0.29
Lesion-associated parenchymal	2.2 (0.0–12.6)	0.0 (0.0–3.1)	− 2.2 (− 0.8 to + 2.8)	**0.005**
abnormalities Normal lung tissue	91.8 (83.5–98.9)	96.0 (86.7–99.1)	+ 4.2 (− 4.6 to + 11.9)	**0.02**
End**- **expiration				
Hypointensity regions	-	7.1 (0.0–24.3)	-	-
Normal lung tissue	-	92.9 (75.7–100.0)	-	-

Data are presented as median (range) of the percentage (%) of the total lung volume

*p* values < 0.05 are in bold

### Quantitative imaging assessment

**Fig. 5:**
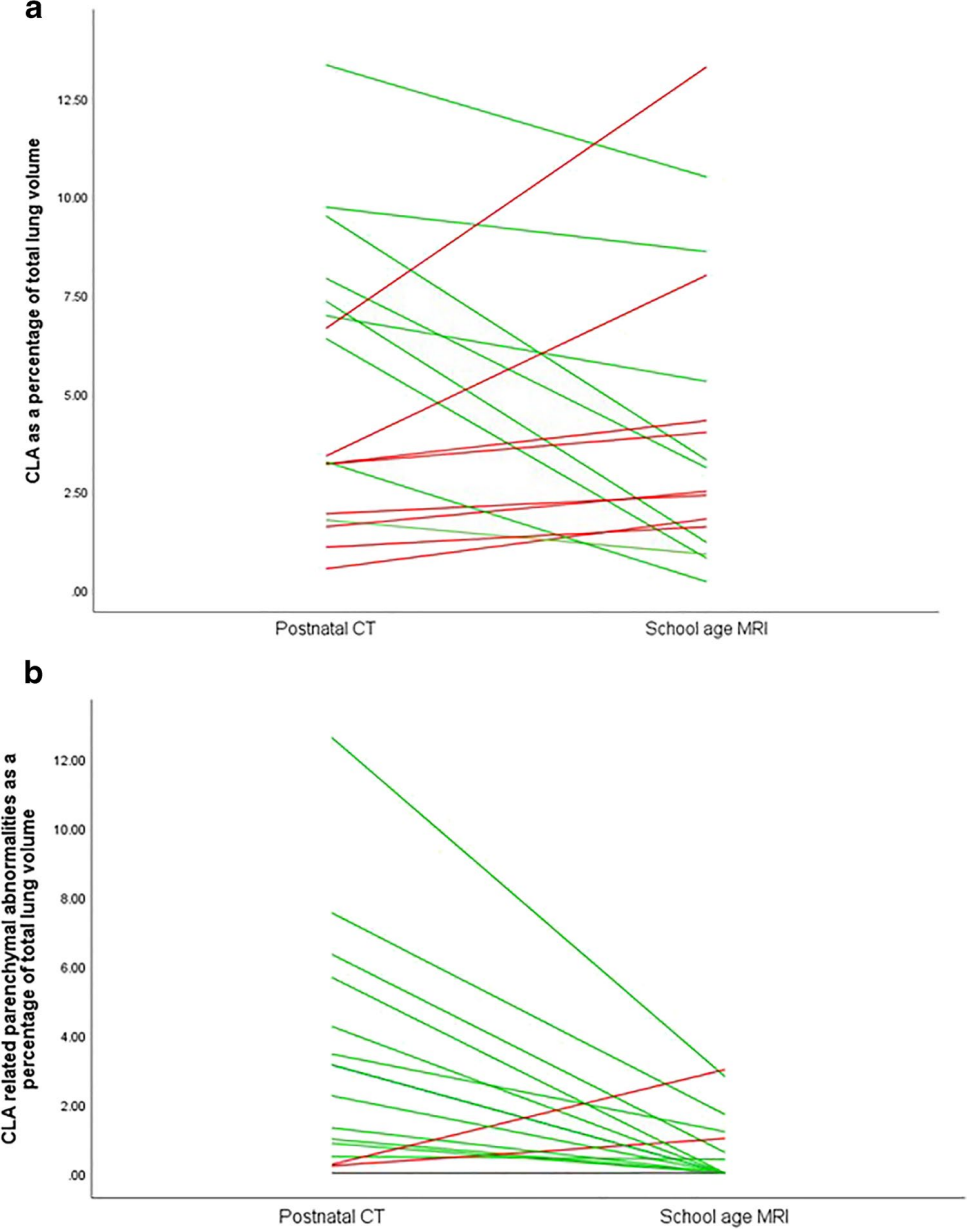
Plot of the changes in (**a**) size of the CLA lesion in relation to total lung volume between postnatal CT and school-age MRI and (**b**) size of lesion-associated parenchymal abnormalities between postnatal CT and school-age MRI. Lines in green indicate a decrease in relative size; lines in red indicate an increase in relative size

Results from the CLAQ and CLAM scoring are shown in Tables 2 and 3. Figure 5a shows an overview of the change in size of the CLA in relation to total lung volume between the CLAQ on postnatal CT and the CLAM on school-age MRI. Comparing the CLAQ and CLAM outcomes in unoperated patients, the median size of the lesion relative to total lung volume remained stable (− 0.9% (range − 6.2% to + 6.7%), *p* = 0.3).

Figure 5b shows the changes in lesion-associated parenchymal abnormalities in relation to total lung volume between postnatal CT and school-age MRI. In 7/17 (41%) patients, the associated parenchymal abnormalities were no longer visible on MRI; in most of these patients, this concerned atelectasis seen during the postnatal period which was absent at school age. One patient had an increase in lesion-associated parenchymal abnormalities. This was a patient with a CPAM on postnatal CT which was scored as BA with associated surrounding parenchymal hypointensity on school-age MRI; hence, this change in classification resulted in a decrease of the volume occupied by the lesion and an increase in the volume of lesion-associated parenchymal abnormalities. Overall, in unoperated patients, the median size of lesion-associated parenchymal abnormalities in relation to total lung tissue decreased (− 2.2% (range − 0.8 to + 2.8%), *p* = 0.005).

On MRI, the most common associated parenchymal abnormality was hypointense lung parenchyma seen on free-breathing images in 35% (6/17) and on expiratory images in 82% (14/17).

In all patients in whom the CLA was surgically resected, only minor scar tissue was observed. One of these patients had a pneumonectomy of the left lung at the age of one week and showed diffuse hypointense lung parenchyma on expiratory MRI (24% of the total lung volume), representing hyperinflation of the remaining right lung.

### Qualitative imaging assessment

**Fig. 6:**
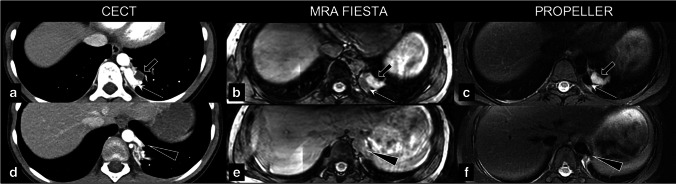
Visualisation of lesion vascularisation on axial contrast-enhanced postnatal CT (**a** and **d**), MRA FIESTA (**b** and **e**), and T2-w PROPELLER (**c** and **f**) in a patient with BPS. Images show venous drainage of the lesion into the hemiazygos vein (thin arrow on **a**, **b**, **c**) and a bronchele (thick arrow on **a**, **b**, **c**) and arterial supply from the aorta descendens (arrow on **d**, **e**, **f**). BPS, bronchopulmonary sequester; CECT, contrast-enhanced computed tomography; MRA FIESTA, magnetic resonance angiography; PROPELLER, periodically overlapping parallel lines with enhanced reconstruction

According to the qualitative imaging assessment included in the structured MRI report, CLA-related abnormalities were best seen on ZTE (airway), PROPELLER (vascularisation), and SPGR expiration (hypointense structures such as cysts, low attenuation regions, and hyperinflation) sequences. Six patients had an MRA FIESTA sequence available; this sequence was rated best to depict abnormal vascularisation in five patients. In one patient, the MRA FIESTA was insufficient due to severe movement artefacts. In two patients with a vascular component of the CLA, no MRA FIESTA sequence was made; however, abnormal vascularisation was sufficiently visualised on the T2-w PROPELLER sequence. An example of the visualisation of lesion vascularisation on postnatal CT compared to school-age MRI is shown in Fig. 6. Qualitative scoring showed acceptable quality for the ZTE sequence, with a median score of ‘above average’ for the visualisation of all lung structures (Table 4). The T2-weighted PROPELLER sequence scored ‘satisfactory’ but did show less noise/artefacts compared to the ZTE sequence.

**Table 4:** Qualitative MRI scoring

	Axial SPGR insp	Axial SPGR exp	T2**-**weighted FS PROPELLER	ZTE	MRA FIESTA
A. Qualitative scoring according to Bae et al					
Fissures	1 (1–1)	1 (1–1)	2 (2–2)	2 (2–2)	*
Vessels	2 (2–2)	2 (2–2)	3 (3–3)	3.5 (3–4)	*
Bronchi	2 (2–2)	2 (2–2)	3 (3–3)	4 (3–4)	*
Noise**/**artefacts	3 (2–3)	3 (2–3)	3 (3–4)	3 (2–4)	*
Overall acceptability	2 (2–3)	2 (2–3)	3 (3–4)	4 (3–4)	*
B. Best sequence to depict CLA-related abnormalities, according to structured MRI report					
Airway	-	-	2	19	-
Cystic lesion	-	2	3	5	-
Solid**/**emphysematous lesion	-	11	5	1	-
Lesion border	-	1	6	10	-
Vascularisation	-	-	15	3	5
Atelectasis	-	1	8	3	-
Low attenuation	-	15	-	1	-
Hyperinflation	-	8	-	-	-

*FS*, fat suppressed; *PROPELLER*, periodically rotated overlapping parallel lines with enhanced reconstruction; *SPGR*, spoiled gradient echo; *UTE*, ultra-short echo time; *ZTE* < zero-echo time. A shows qualitative scoring of the MRI sequences according to Bae et al (European Radiology 2020). *No qualitative scoring was performed on the MRA FIESTA sequence. Data is presented as median (interquartile range) B shows the qualitative score according to the structured MRI report; data are presented as the number of patients in whom this sequence was scored as best depicting CLA-related abnormalities (note, not all structures were present in all patients)

## Discussion

In this study, we have used non-contrast MRI for the long-term follow-up of CLA patients and describe our findings in a cohort of school-age CLA patients in comparison to postnatal CT. We show that MRI is able to identify all CLA-related lung structures. In addition, we show the development of unoperated CLA over time.

In 41% of the unoperated patients, the radiological appearance, and therefore radiological diagnostic interpretation of the CLA, was different between postnatal and school age. The different appearance of the CLA found in this study has multiple possible explanations. First, this could be related to an actual change in the CLA. Examples are the disappearance of cystic tissue or the accumulation of mucus in a BA changing its appearance into a bronchocele, which increases visibility on an MRI image compared to an airway without mucus, as we saw in two patients [25]. Second, the change in appearance can be related to age-related growth of body size making certain lung structures, such as the airways, better visible on MRI images. Furthermore, growth-related changes in the appearance of CLA can also be related to progressive hyperinflation of lung parenchyma due to collateral ventilation, appearing as hypointense regions on MRI which were not initially identified on postnatal CT [26–28].

A third explanation for the different appearance of some of the CLA is the difference in imaging modality used between postnatal and school-age follow-up. On one hand, CT has a higher image resolution compared to MRI. On the other hand, our MRI protocol in cooperative school-age children offers the possibility to perform end in- and expiratory scans, showing the lung at residual volume (RV) with a superior contrast-to-noise ratio compared to images taken at functional residual capacity (FRC), as is the case in postnatal CT imaging. These RV images are more sensitive to visualise hypointense regions, such as CLO and BA, than CT.

On average, in unoperated patients, the size of the lesion in relation to total lung volume did not change between postnatal and school-age follow-up. Although this finding corresponds to our experience from clinical practice, no long-term follow-up studies have reported on the development of CLA over time. The stable relative size of the lesion, in combination with spirometry outcomes within the normal range, could justify the wait-and-see approach in asymptomatic CLA patients [6]. These findings are in line with a study describing normal lung function in CLA patients that did not undergo surgery [29]. In addition, studies comparing prenatal screening to postnatal follow-up frequently describe regression of CLA. But our findings show that this regression of CLA does not occur after the postnatal period [30].

We also found a decrease in lesion-associated parenchymal abnormalities in relation to total lung volume between postnatal and school-age follow-up. The relative decrease was most often related to atelectasis seen on postnatal CT that decreased or disappeared on school-age MRI. This atelectasis seen on postnatal CT could be related to either local compression of the primary lesion on the surrounding parenchyma that decreases over time, indicating possible decreased impact of the CLA on the normal lung tissue over time. In addition, this could also be related to the young age of the patients and thereby prolonged periods of supine position, or anaesthesia [31, 32]. Both reasons show that atelectasis on postnatal imaging may not be related to the clinical condition of the patient.

Our qualitative scoring revealed which MRI sequences are best suitable for the visualisation of CLA. First, the best structural visualisation of the CLA was achieved with the ZTE and SPGR expiratory sequences. Second, hyperdense regions, such as atelectasis, were best visualised on the T2-w PROPELLER sequence. Third, vascularisation was best visualised on the MRA sequence, but could also be visualised on T2-w PROPELLER sequences. These findings show that contrast enhancement is not needed for the follow-up of CLA when using MRI. Thereby, patient compliance to the examination could be increased by omitting the need for intravenous access and possible concerns related to gadolinium deposition [33].

To our knowledge, this is the first study using non-contrast MRI for the long-term follow-up of CLA. A recent study by Zirpoli et al compared postnatal contrast-enhanced CT to postnatal non-contrast MRI. MRI was found to be comparable to CT for the visualisation of all CLA-related lung structures, except for vascularisation [14]. A study by Kellenberger et al compared postnatal contrast-enhanced CT to contrast-enhanced MRI, and although this study describes comparable findings on the two modalities, contrast enhancement is described as indispensable [15]. In contrast, we found that MRI was able to identify CLA-related vascular abnormalities in all patients. However, our study was spirometer controlled, conducted in an older population, and an MRA sequence was added to our MRI protocol, specifically to image the vasculature. Both studies also did not include an ultra-short echo time sequence in their MRI protocol, which from our qualitative analyses proved best for CLA visualisation. In addition, recent studies have shown these ultra-short echo time sequences to be ideal for postnatal imaging due to superior image resolution and the additional benefit of these sequences being silent on some MRI systems and therefore less burdensome for young children [34–36]. Further research is needed to standardise postnatal MRI protocols for CLA between MRI vendors, including the addition of the most recently developed sequences.

Our study has some limitations. First, this was a single-centre study with a small number of study participants. Second, we did not have school-age CT available for direct comparison. Another point that is not addressed in our study is the ability of MRI to detect possible development of malignancy within CLA, for which our population size is too small considering the extremely low incidence [10, 37]. As a matter of fact, the radiological characteristics of malignant deterioration of CLA are not well defined [38].

In conclusion, this is the first study describing the use of non-contrast MRI for the follow-up of CLA. Based on our study, MRI is able to identify the most important CLA-related lung abnormalities without the use of contrast enhancement or sedation. We also show that the radiological appearance of a large proportion of the unoperated CLA changes over time, related to actual changes in the CLA, growth of the patient, and imaging modalities used. Further research is needed to validate our results in a larger cohort and to compare school-age MRI to school-age CT. Such a study will allow introduction of MRI as safe imaging method alternative for chest CT for the long-term follow-up of paediatric CLA patients.

## Supplementary information

Below is the link to the electronic supplementary material.

Supplementary file1 (PDF 209 KB)

## Abbreviations

ATS: American Thoracic Society
BA: Bronchial atresia
BC: Bronchogenic cyst
BPS: Bronchopulmonary sequestration
CLA: Congenital lung abnormalities
CLAM: Congenital lung abnormalities quantification on MRI
CLAQ: Congenital lung abnormalities quantification on CT
CLO: Congenital lobar overinflation
CPAM: Congenital pulmonary airway malformations
CT: Computed tomography
ERS: European Respiratory Society
FEV_1_: Forced expiratory volume in 1 s
FRC: Functional residual capacity
FS: Fat suppressed
FVC: Forced vital capacity
GLI: Global Lung Function Initiative
PD-w: Proton density weighted
PROPELLER: Periodically rotated overlapping parallel lines with enhanced reconstruction
MRA: Magnetic resonance angiography
MRI: Magnetic resonance imaging
RV: Residual volume
SPGR: Spoiled gradient recalled echo
T2-w: T2 weighted
ZTE: Zero echo time

## Declarations

### Guarantor

The scientific guarantor of this publication is dr. P Ciet, Erasmus MC- Sophia, p.ciet@erasmusmc.nl.

### Statistics and biometry

No complex statistical methods were necessary for this paper.

### Methodology

- prospective
- cross-sectional study
- performed at one institution
