# Supplementary material for: Congenital lung abnormalities on magnetic resonance imaging: the CLAM study
Source: Eur Radiol. 2023 Feb 24;33(7):4767–79. doi: 10.1007/s00330-023-09458-7 (PMC10290040; doi:10.1007/s00330-023-09458-7)
Supplement: Supplementary file 1 — Supplementary file1 (PDF 209 KB) [file 330_2023_9458_MOESM1_ESM.pdf]

Online supplement 1: MRI protocol

Sequence	SPGR inspiration/ expiration	FS T2 weighted PROPELLER	ZTE	MRA Fiesta
**Acquisition plane**	Sagittal	Axial	Axial	Coronal
**TR/TE (ms)**	1.5/0.6	*/73	1.1/0	3.6/1.1
**flip angle (°)**	2	90/120	2	50
**RF**	Selective	Selective	Non-selective	Selective
**In-plane matrix**	120x120	340x340	200x200	220x220
**k-space trajectory**	Cartesian	BLADES	Radial	Cartesian
**In-plane Field-of-view (FOV)**	36	34	30	40
**RecFOV**	0.75	-	-	-
**Actual voxel resolution (mm^3)**	3.0x3.0x3.0	1.0x1.0x3.5	1.5x1.5x1.5	1.8x1.8x1.8
**Slices**	90 (as in VIBE)	50-70	200	60-80
**Slice thickness**	3.0	3.5	1.5	1.8
**Receiver bandwidth (KHz)**	90	83.33	62.5	100
**Parallel imaging**	ARC (1.5x1.2)	3.0	None	Asset (2.0)
**Number of average**	1	2.0	2.5	1
**No. of spokes per segment**	-	14	512	150-180 ms
**Physiological triggering**	BH	Prospective pencil beam navigator	Prospective projection navigator	Respiratory gated and cardiac triggered
**Scan time (sec) RR=20**	+/- 6sec	+/- 5 min	+/- 6 min	+/- 6 min

Online supplement 1: MRI protocol. FOV; Field Of View, FS: fat suppressed, NEX; Number of Excitations, PROPELLER; Periodically Overlapping ParalLEL Lines with Enhanced Reconstruction, RF; radio frequency, SPGR; Spoiled Gradient Echo Sequence, TE; Echo Time, TR; Repetition Time. * TR chosen depended on the respiratory frequency of the patient.

Online supplement 2:

Online supplement 2A: Structured CT report

Structured CT report Congenital Lung Abnormalities

Scan protocol:

☐ Inspiration ☐ Volumetric Slice thickness: mm

☐ Expiration ☐ Volumetric Slice thickness: mm

Contrast enhancement:

☐ None

☐ Venous phase

☐ Arterial phase

A. Findings:

Location & extent:

	RUL	RML	RLL	LUL	LLL
Extent					
1= ⅓ lobe 2= ⅔ lobe 3=complete lobe					

Airway:

☐ Normal connection

☐ Bronchomalacia

☐ Atresia

B. Lesion:

Cystic tissue:

- Structure:

  ☐ Dominant cyst ☐ Cluster of multiple uniform cysts

- Average cyst size: mm
- Size of largest cyst: mm
- Content:

  ☐ Air-filled

  ☐ Fluid-filled

  ☐ Air-Fluid levels

Solid tissue:

	RUL	RML	RLL	LUL	LLL
Extent					
1= ⅓ lobe 2= ⅔ lobe 3=whole lobe					

- Density:

  ☐ Homogeneous

  ☐ Heterogeneous

C. Border:

☐ Well-defined ☐ Ill-defined

D. Vascularization:

Arterial:

☐ Normal pulmonary

☐ Aberrant :

Number & Size: Origin:

Venous:

☐ Normal pulmonary

☐ Aberrant :

Number & Size: Origin:

E. (Surrounding) tissue:

	RUL	RML	RLL	LUL	LLL
Atelectasis					
Low-attenuation					
Hyperinflation					
1= ⅓ lobe 2= ⅔ lobe 3=whole lobe					

☐ Mass effect

☐ Mediastinal shift

☐ Mediastinal herniation

F. Conclusion

☐ CPAM

☐ BPS

☐ Hybrid lesion

☐ CLO

☐ BA

☐ BC

Online supplement 2B: Structured MRI report

Structured MRI report Congenital Lung Abnormalities

A. Findings:

Location & extent:

	RUL	RML	RLL	LUL	LLL
Extent					
1= ⅓ lobe 2= ⅔ lobe 3=complete lobe					

Airway:

☐ Normal connection

☐ Bronchomalacia

☐ Atresia

☐ Not clearly visible

On which sequence was the airway best visualized:

☐ SPGR inspiration

☐ SPGR expiration

☐ T2-w PROPELLER

☐ ZTE

☐ Other, namely…

B. Lesion:

Cystic tissue:

- Structure:
  - ☐ Dominant cyst
  - ☐ Cluster of multiple uniform cysts
  - ☐ Not clearly visible
- Average cyst size: mm
- Size of largest cyst: mm
- Content:
  - ☐ Air-filled
  - ☐ Fluid-filled
  - ☐ Air-Fluid levels
  - ☐ Not clearly visible

On which sequence was the cyst(s) best visualized:

☐ SPGR inspiration

☐ SPGR expiration

☐ T2-w PROPELLER

☐ ZTE

☐ Other, namely…

Solid tissue:

	RUL	RML	RLL	LUL	LLL
Extent					
1= ⅓ lobe 2= ⅔ lobe 3=whole lobe					

- Density:
  - ☐ Homogeneous
  - ☐ Heterogeneous

On which sequence was the solid tissue best visualized:

☐ SPGR inspiration

☐ SPGR expiration

☐ T2-w PROPELLER

☐ ZTE

☐ Other, namely…

C. Border:

☐ Well-defined ☐ Ill-defined ☐ Not clearly visible

On which sequence was the lesion border best visualized:

☐ SPGR inspiration

☐ SPGR expiration

☐ T2-w PROPELLER

☐ ZTE

☐ Other, namely…

D. Vascularization:

☐ Normal pulmonary

☐ Aberrant :

Number & Size: Origin:

On which sequence was the vascularization best visualized:

☐ SPGR inspiration

☐ SPGR expiration

☐ T2-w PROPELLER

☐ ZTE

☐ MRA

E. (Surrounding) tissue:

	RUL	RML	RLL	LUL	LLL
Atelectasis					
Low-attenuation					
Hyperinflation					
1= ⅓ lobe 2= ⅔ lobe 3=whole lobe					

☐ Mass effect

☐ Mediastinal shift

☐ Mediastinal herniation

On which sequence was atelectasis best visualized:

☐ SPGR inspiration

☐ SPGR expiration

☐ T2-w PROPELLER

☐ ZTE

☐ Other, namely…

On which sequence was low-attenuation best visualized:

☐ SPGR inspiration

☐ SPGR expiration

☐ T2-w PROPELLER

☐ ZTE

☐ Other, namely…

On which sequence was hyperinflation best visualized:

☐ SPGR inspiration

☐ SPGR expiration

☐ T2-w PROPELLER

☐ ZTE

☐ Other, namely…

F. Conclusion

☐ Post- surgical CLA

☐ CPAM

☐ BPS

☐ Hybrid lesion

☐ CLO

☐ BA

☐ BC

Online supplement 3: Qualitative scoring method

**Depiction of fissures**	1. Unacceptable (invisible interlobar fissure)<br>2. Fair (blurred interlobar fissure)<br>3. Good (visible interlobar fissure)
**Depiction of intrapulmonary vessels**	1. Unacceptable (invisible peripheral pulmonary vessels)<br>2. Poor (barely visible peripheral pulmonary vessels)<br>3. Fair (visible peripheral pulmonary vessels)<br>4. Good (visible peripheral pulmonary vessels with clear margin)<br>5. Excellent (visible peripheral pulmonary vessels with clear margin)
**Depiction of bronchi**	1. Unacceptable (indistinguishable lobar bronchial walls)<br>2. Poor (visible lobar bronchial walls with <10 visible segmental bronchial walls)<br>3. Fair (visible lobar bronchial walls with >10 visible segmental bronchial walls)<br>4. Good (visible lobar bronchial walls with >10 visible segmental bronchial walls, with few visible sub/segmental bronchial walls)<br>5. Excellent (visible sub-subsegmental bronchial walls)
**Image noise/artifacts**	1. Unacceptable<br>2. Above average noise/artifacts<br>3. Average and acceptable<br>4. Less than average noise/ artifacts<br>5. Minimum of no noise/artifacts
**Overall acceptability**	1. Unacceptable<br>2. Suboptimal<br>3. Satisfactory<br>4. Above average<br>5. Superior

Online supplement 3: Qualitative scoring method adjusted from Bae *et al*. (European Radiology 2020)
